# The role of hydrodynamics in collective motions of fish schools and bioinspired underwater robots

**DOI:** 10.1098/rsif.2023.0357

**Published:** 2023-10-25

**Authors:** Hungtang Ko, George Lauder, Radhika Nagpal

**Affiliations:** ^1^ Mechanical and Aerospace Engineering, Princeton University, Princeton, NJ, USA; ^2^ Organismic and Evolutionary Biology, Harvard University, Cambridge, MA, USA; ^3^ Computer Science, Princeton University, Princeton, NJ, USA

**Keywords:** fluid stigmergy, collective behaviour, fluid mechanics, fish school

## Abstract

Collective behaviour defines the lives of many animal species on the Earth. Underwater swarms span several orders of magnitude in size, from coral larvae and krill to tunas and dolphins. Agent-based algorithms have modelled collective movements of animal groups by use of *social forces*, which approximate the behaviour of individual animals. But details of how swarming individuals interact with the fluid environment are often under-examined. How do fluid forces shape aquatic swarms? How do fish use their flow-sensing capabilities to coordinate with their schooling mates? We propose viewing underwater collective behaviour from the framework of *fluid stigmergy*, which considers both physical interactions and information transfer in fluid environments. Understanding the role of hydrodynamics in aquatic collectives requires multi-disciplinary efforts across fluid mechanics, biology and biomimetic robotics. To facilitate future collaborations, we synthesize key studies in these fields.

## 1. Introduction

**Figure 1.**
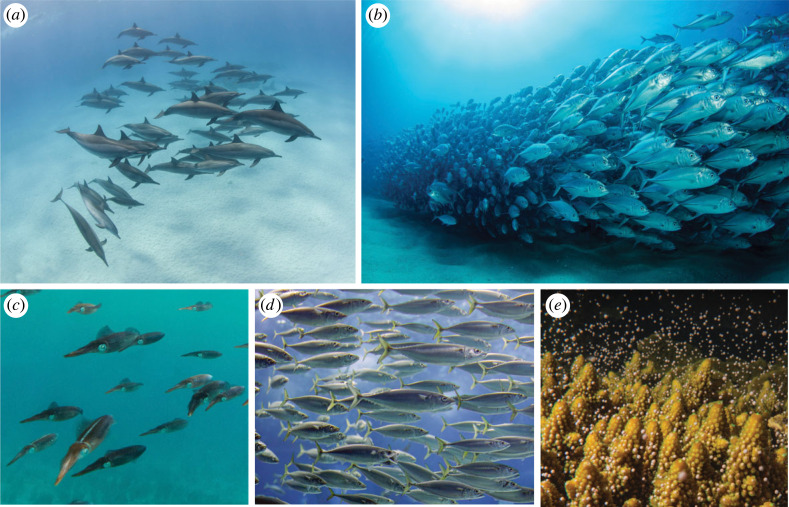
Underwater swarms in nature span several orders of magnitude, from (*a*) dolphins and (*b*) giant trevally on the metre scale, to (*c*) squid and (*d*) mackerel on the centimetre scale, to (*e*) coral larvae on the millimetre scale. Images obtained through Education licences from Adobe Stock.

Animal collective behaviour has long been a fascination and inspiration for humans [1]. Underwater swarms span several orders of magnitude in size, from clouds of coral larvae on the millimetre scale to pods of dolphins on the metre scale (figure 1). In groups, animals achieve functions that individuals cannot achieve, forming a whole greater than the sum of its parts. Coordination between animals in large groups requires unique strategies [2]. Biological organisms have limited sensing capabilities and individuals do not know the precise states (location, velocity, orientation, etc.) of other group members. How do they respond to the sensory information available to them in a way that gives rise to emergent behaviour on the collective level?

**Figure 2.**
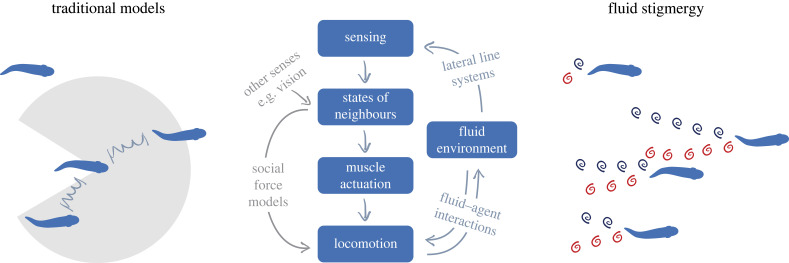
Different constructs of swarm models. Agent interactions in traditional models depend on relative distance and locations, sometimes considering vision and a limited field of view. The perspective of fluid stigmergy includes the fluid environment and emphasizes fluid–agent interactions and coordination strategies based on flow-sensing.

This question has been investigated by the use of agent-based models, which simulate emergent group behaviour based on assumed rules for individual agents. Such models often impose a social force that either attracts or repels an individual to its neighbour based on their distance as if they were connected via a virtual spring (figure 2). Social forces like this are a crucial element of the Boids model [3] and the Viscek model [4] as they facilitate generating coherent movements in simulations. Despite its effectiveness, social force is only a reduced-order approximation of animal behaviour, and limitations of animals’ sensing and locomotion are rarely considered (figure 2). While social force may be fitted from experiments, there is no guarantee that its form and magnitude apply to swarms of different sizes, densities and speeds: the social force is itself an emergent property.

Going beyond such simplifications, the sensory systems of swarming animals must be considered. Aquatic organisms perceive the world through synthesizing a wide range of sensory information, including visual, hydrodynamic, proprioceptive, electric and magnetic cues. Classical models such as [5,6] were among the first to consider a limited field of view. Others limited the number of perceived neighbours [7], or designed agent behaviours entirely based on vision [8]. The roles of sensing modes other than vision have rarely been considered in existing models of swarms. However, many aquatic species have limited vision or live in dark environments. These organisms cannot sense a neighbour a few body lengths away as assumed in vision-based models. They must rely on other sensory inputs like hydrodynamic cues and use coordination strategies that are more short-range and environment-dependent.

A crucial aspect often neglected in fish school models is the effect of the fluid environment (figure 2). The concept of environment-mediated coordination dates back more than 60 years. *Stigmergy* was initially coined to describe the collective construction of termite mounds [9–11]. Instead of exchanging information explicitly with each other, termites communicate and coordinate indirectly by leaving and sensing traces in the environment. A similar principle is also used by ants to follow trails [12], by paper wasps to construct nests [13], and by honeybees to locate their queens [14–16]. Recently, stigmergy has been extended beyond social insects to bacterial colonies [17,18], spatio-temporal patterns of animal territories [19], cognition [20,21] and swarm robots [22–24].

In this perspective, we propose to view aquatic collective systems through the lens of *fluid stigmergy*, which urges considerations of the dynamic fluid environment and the information it carries. How are movements of underwater collectives affected by fluid forces? How can individuals in groups use hydrodynamic information for coordination? The wake behind a fish carries information about the individual’s state, much like the ants’ pheromone trails. In addition, spatial–temporal features of the vortex street are advected and diffused according to the same principle that transports airborne pheromones of social insects like honeybees [14–16]. This flow information is critical for underwater collective movements, especially for animals with limited long-range sensing capabilities. How stigmergy facilitates collective behaviour likely depends on the scale and the Reynolds number (*Re*), which defines the ratio between inertia and viscous effects. For low *Re* organisms such as bacteria (*Re* < 1), disturbances diffuse almost instantaneously but may be sensed from afar. For larger *Re* swimmers such as schooling fish (*Re* = 10^4^−10^6^), vortices retain their structures longer but their influence is short-ranged. In this regime, while a swimming fish is difficult to detect a few body lengths away, its follower who swims through vortices may obtain rich information about its states. Indeed, it has been demonstrated that with only hydrodynamic information, a blindfolded fish can school with its neighbours [25]. The relative role of vision and flow sensing depends on the species and their native habitats. But *fluid stigmergy* (figure 2) is likely used, at least partially, by all schooling species.

Compounding the complexity of underwater coordination are the physical fluid–agent interactions. Passive agents such as dead fish or granular materials can exhibit non-intuitive behaviours under fluid forces. Just as hydrodynamic interactions can influence the collective behaviour of microscopic organisms [26], repulsion and attraction between a pair of fish can emerge in the absence of social interactions and social forces [27,28]. In the wake behind an obstacle, a dead fish can undulate its body and keep up with the flow as if it were alive and swimming [29]. Recently, it was shown that rheotaxic behaviour may not require any sensory feedback either [30]. These hydrodynamic factors led to the classical conjecture posed by Weihs & Lighthill, which stated that a diamond formation of fish schools is optimal because it would lead to constructive vortex interactions and allow schooling individuals to save energy [31,32]. In addition, Lighthill surmised that such a formation is stable—if a fish deviates from the diamond formation, fluid forces will push it back to its original position. Whether a diamond formation is truly hydrodynamically optimal and whether fish schools in nature prefer such a formation remains an open question. However, the key notion stands: hydrodynamic interactions could lead to self-organizing effects in spite of social interactions. Coupling with these effects, movements of active agents can give rise to unexpected collective behaviours [33].

Fluid–agent interactions affect not only natural swarms but also underwater robot collectives. Recently, swarm robotics have received increasing interest because they benefit from low cost, versatility and robustness [34,35]. Most current robot swarms are controlled using social force models. Taking advantage of technologies such as WiFi, Bluetooth and centralized tracking systems, terrestrial robots can perceive neighbours' locations better than biological organisms, making coordination simpler. However, underwater robots do not enjoy the same benefits. Electromagnetic waves attenuate rapidly and sonar suffers from low bandwidth and high latency [36]. As such, low-cost and low-power underwater swarms must rely on visual and hydrodynamic cues much like schooling fish. Recently, a vision-based underwater robot swarm has been demonstrated to achieve collective behaviours such as milling [37]. Future designs that include a flow sensing system may enable robot swarms to save energy by taking advantage of the fluid–agent interactions and using fluid stigmergy for coordination.

In this review, we discuss findings and methods in fluid mechanics, biology and robotics with the perspective of fluid stigmergy. We limit our scope to underwater systems since most aerial animals such as starlings possess acute vision and unmanned aerial vehicles often rely on wireless communication. In the next section, we start by examining the fluid field around fish schools, discussing what hydrodynamic information a swimming fish leaves behind, and how swarming individuals may interact with each other through fluid forces. In §3, we discuss what is known about how fish sense their fluid environment. In §4, we review robotic platforms with flow-sensing capabilities and summarize what behaviours flow-sensing enables. Finally, in §5, we highlight key challenges and opportunities for understanding underwater systems in the future.

## 2. Understanding fluid flow

To start, we must understand the fluid mechanics of fish schools. What is the flow signature a fish leaves behind? How is that information preserved and propagated to another fish nearby? What schooling formations are hydrodynamically efficient and stable? The complex interaction between fish schools and water flow has been an ongoing research topic within the fluid mechanics community, and progress has been made using both experiments and simulations [38–41,42, ch. 3].

**Figure 3.**
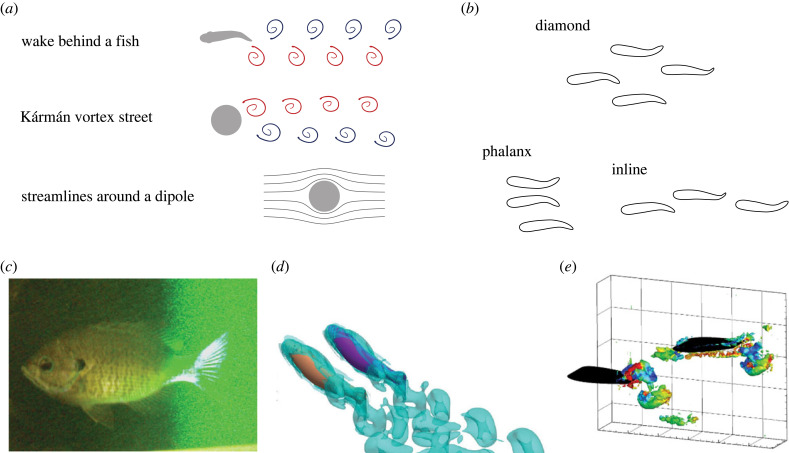
(*a*) Fluid fields behind a swimming fish, behind an obstacle, and around a dipole. Red vortices rotate clockwise while blue vortices rotate counterclockwise. (*b*) Hydrodynamic arguments support either a diamond formation, a phalanx formation or an inline formation. (*c*) The three-dimensional fluid field around a swimming fish can be characterized by tomographic PIV (permission from [43]), and hydrodynamic interactions between a pair of fish can be studied using (*d*) fluid simulations (permission from [44]) and (*e*) hydrofoil experiments (permission from [45]).

When a fish swims through the water, it leaves two rows of vortices behind (figure 3*a*). Each vortex forms as the tail swings from one side to the other and sheds when it reverses course. Vortices alternate in their direction (clockwise versus counterclockwise). As they travel downstream, vortices dissipate energy and break down into smaller eddies. The rate of energy dissipation and the angle of the vortex street depends on various factors such as the swimming velocity and the flow regime (turbulent versus laminar). The width and wavelength of the wake decrease as the tail-beat frequency increases and smaller vortices develop when the frequency is low [46]. The flow signature behind a swimming fish has a strong frequency component. Recently, it has been shown that fluctuations of the fluid field preserve information about a fish’s relative position, phase differences and tail-beat frequency [44].

The simplest way to generate a wake is by placing an obstacle in a current. The flow over a fixed blunt object produces alternating vortices, called the Kármán vortex street [47] (figure 3*a*). This flow pattern can be found behind pillars under bridges or behind rocks in rivers. The rotation directions of the vortices in Kármán vortex street are the opposite of those in a fish’s wake. Therefore, the wake of a fish is also referred to as the *reverse* Kármán vortex street. Fundamentally, the arrangement of the wake vortices relates to whether the object is producing drag (pillars) or thrust (fish) [48]. A key parameter that determines the force balance is Strouhal number, *St* = *fL*/*U*, or frequency *f* non-dimensionalized by the length scale *L* and flow velocity *U*. The emergent vortex-shedding frequency of a Kármán vortex street behind a cylinder results in *St* ≈ 0.2−0.3. Remarkably, for fish swimming at higher speeds, *St* is also in this same range [38,39,41,49]. Now defined with tail-beat amplitude as the length scale and swimming speed as the velocity scale, the non-dimensional tail-beat frequency *St* ≈ 0.3, with most aquatic species ranging narrowly within 0.2–0.4 [38,39,41,49]. This relationship can be used to estimate an undulatory swimmer’s speed based on its beating amplitude and frequency.

Fish wakes can be further abstracted using the potential flow theory in fluid mechanics [50]. Flow around a cylinder can be expressed analytically as a dipole. Since the theory neglects viscous effects, the flow around a dipole does not shed periodic vortices and only approximates the time-averaged fluid field (figure 3*a*). To better mimic the wake behind a fish, in both experiments and simulations, researchers use oscillating spheres and dipoles. However, even in such a simplified setting, complexity quickly arises when there is more than one body in the fluid domain. Placing a sensor or a fish body (dead or alive) will instantaneously change the flow field, including the structure of the vortex street. Indeed, the hydrodynamic interactions between a fish body and its own fins can already lead to non-intuitive results [51,52]. To study what a fish senses from its neighbour’s wake, it is crucial to include multiple fish in the fluid field.

The hydrodynamics of fish schools remain an active area of research. The elegant conjecture that Weihs and Lighthill proposed about the diamond formation (figure 3*b*) has been challenged with animal experiments [53–55], and numerical simulations [27,56,57]. Weihs’ argument for a diamond pattern is based upon constructive vortex interaction in directions both parallel and perpendicular to the swimming directions, assuming the formation remains unchanged. However, a recent study suggested that the constructive fluid–structure interaction is more reliable in the fish’s lateral direction [56]. Therefore, the side-by-side ‘phalanx’ formation is more efficient than the diamond formation (figure 3*b*). This claim is supported by experiments of schools of red nose tetra *Hemigrammus bleheri*, who form increasingly one-dimensional patterns as swimming speed increases. This phalanx formation allowed the tetras to reach faster swimming speeds with lower tail-beat frequencies [55,58]. However, experiments with five species of rainbowfish (*Melanotaenia*) arrived at a conflicting conclusion, showing more alignment along the swimming direction (figure 3*b*) as their speed increases [59]. To add to the puzzle, using two-dimensional simulations with periodic boundaries, research suggests that hydrodynamic benefits are easier to obtain than previously thought [57]. Fish schools are more efficient than individuals regardless of the formations (diamond, rectangular, side-by-side and inline) [57]. It is unclear if and how the preferred schooling formations depend on species, especially regarding the relative importance of the senses (hydrodynamic versus vision) for coordination. Perhaps more critically, whether fish can maintain fixed formations for prolonged periods at all is questionable. Both early literature and empirical observations suggest that fish school formations are transient and dynamic [60].

How do fluid forces push fish in and out of transient formations? Investigating the hydrodynamic stability of various formations has gained considerable interest. For a pair of hydrofoils in tandem with a constant frequency, amplitude and spacing, two stable swimming speeds exist [61]. The two emergent modes, fast and slow modes, correspond to when the flappers are out-of-phase and in-phase, respectively. If the spacing is allowed to vary spontaneously, configurations with synchronized phases (slow modes) are always preferred and are hydrodynamically stable—fluid force will restore their states if perturbed [62]. As more degrees of freedom are added, a stability diagram may be obtained [63]. When the frequencies of the two hydrofoils are matched, stable configurations exist for a range of heaving amplitudes. Intriguingly, *an oscillatory mode* also exists in the regime where the follower heaves wider but less frequently. In this mode, fluid forces orchestrate the periodic movement of the follower, at times closing the gap and at others failing to keep up. These behaviours have been observed in numerical simulations where the swimmers deform their bodies like fish and are allowed to rotate [64,65]. Oscillatory modes have also been found for two simulated fish starting from side-by-side formations [66]. For up to four swimmers, researchers have discovered more than a dozen equilibrium formations, including the diamond formation that Weihs suggested [67]. However, simulations in [67] are two-dimensional, and swimmers can only move freely along the streamwise direction. Recent studies using three-dimensional simulations [27] and hydrofoils free to move in both directions [28] showed that very few formations are truly stable.

These studies of hydrodynamic performance and stability of fish schools have generated considerable insight into schooling strategies. The fact that stable equilibrium states are rare suggests a stability–manoeuvrability trade-off similar to bird flight [68,69]: if fish are attracted strongly to equilibrium positions by fluid forces, they will lose the ability to change directions rapidly. However, while a bird can engineer the stability landscape by changing its body conformation and movement frequency, the stability landscape of a fish is determined by the movements of its schooling mates! The scarcity of stable formations also suggests that realistic formations of underwater swarms must be ever-changing (unless deliberate efforts and costs are paid to maintain them). This, however, does not rule out the possibility of identifying any patterns in transient schools since dynamic oscillatory modes are also likely.

To study the hydrodynamics of fish schools, a wide range of experimental and numerical tools may be adopted. Particle image velocimetry (PIV) is widely used in experiments to characterize fluid flows around live fish [70–73]. The technique has been extended to study the three-dimensional flow field around individual swimming fish (figure 3*c*) [43,74–76]. PIV has also been applied to multiple interacting hydro-foils (figure 3*d*) [28,45,61,62]. Numerically, researchers have used methods such as direct numerical simulations with immersed boundaries (figure 3*e*) [27,44,56,66,67,77–79], multi-particle collision dynamics model [57], and potential flow based solvers [50,80]. However, most existing simulations prescribe the movements of the fish bodies, and the force balance around a fish is not always strictly enforced. As these computational techniques advance, realistic simulations with multiple interacting individuals may soon be achieved. Further, interfacing fluid simulations with agents that can actively sense and react to their fluid surroundings is a fruitful direction for future work.

## 3. Flow-sensing in fish schools

The sense of touch (mechano-sensation) is universal for animals both on land and underwater, but the form of mechanical forces differs significantly. Aquatic animals adopt unique strategies to sense fluid flows around them and can sense ‘touch at a distance’ [42]. Their mechano-sensory organ, the lateral line system, senses perturbations across a wide range of frequencies, from steady currents to acoustic waves. Lateral line systems have attracted significant research since the seventeenth century and have been reviewed excellently by Coombs and co-workers [42,81]. Here, we focus on the mechanical properties of lateral line systems as they relate to the function of sensing fluid movement and their role in implicit coordination.

**Figure 4.**
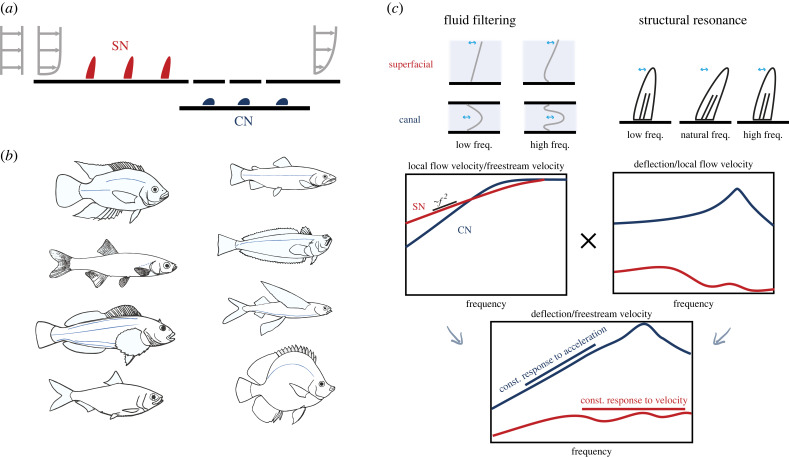
(*a*) Superficial neuromasts (SN, red) and canal neuromasts (CN, blue) and their relative relationship with the boundary layer flow. Grey arrows indicate fluid velocity. (*b*) Interspecies diversity in trunk canal placements (adapted from [82]). (*c*) Frequency response of SN and CNs (adapted from [42]).

Lateral line systems can be divided into two subgroups: superficial neuromasts (SN) and canal neuromasts (CN) (figure 4*a*). They consist of dome-shaped epithelial structures (cupula) covering hair cells that deflect under fluid flows. Both types of neuromasts sense fluid shear to infer flow intensity. SNs protrude from the surface and directly sense the flow velocity in their vicinity. CNs sense fluid flow within the lateral line canal. Since the flow in the canal is dominated by viscous effects, its speed is proportional to the pressure difference between pores that are connected to the exterior environment. Therefore, CNs effectively sense the pressure drop in the external flow. SNs often locate in proximity to CNs [83], and canals can be found both on the head (cranial canals) and along the body of a fish (trunk canal). Canals are denser on the head, surrounding the eye and extending from the cheek to the jaw (figure 4*b*), providing hydrodynamic velocity information from all directions. The difference among those signals may be used to infer the direction of flow or to locate prey [82,83]. With only two types of fluid sensors available to them (SNs and CNs), how do fish arrange them to maximize their sensing capabilities? The placement and relative abundance of SNs and CNs are extremely diverse (figure 4*b*) [42, ch. 2; 81, ch. 10; 82–84], but it has been noted that fish living in fast and turbulent waters tend to have only a few or no SNs [82]. This trend may be explained by considering the boundary layer that forms around the body of a fish (figure 4*a*) [73,85–87]. The boundary layer is thinner for fast and turbulent flows. In these environments, the boundary layer can shrink to thicknesses comparable to the size of neuromasts, and the protrusion of SNs would significantly alter the flow. Sheltered by the canal, the performance of CNs is less affected by boundary layers in fast currents.

Hydrodynamic information produced by a moving fish is intrinsically periodic. While a single tail beat only creates small disturbances that dissipate rapidly with distance and time, periodic tail beats produce pressure waves that can travel much farther than the scale of the animal. Indeed, swimming fish create acoustic waves! It is unclear if such a wave is strong enough to be distinguished from background ocean noise, but research has long shown that fish can use lateral line systems to sense low-frequency vibrations [42]. In fact, during most of the nineteenth and twentieth centuries, lateral lines were considered auditory organs, and researchers have investigated how neuromasts respond to oscillatory flow [42]. Like trees and buildings, neuromasts have natural frequencies, and their oscillatory movements depend on inertia and rigidity. As a result, signals within a certain frequency range are amplified, while others are attenuated (figure 4*c*). Cave-dwelling populations of Mexican cavefish (*Astyanax mexicanus*) have taller SNs and are more responsive to lower frequency stimuli compared with stream-dwelling populations that possess functional visual systems [88]. Fluid properties also affect neuromasts’ frequency response. As the perturbation frequency increases, inertial effects begin to dominate over viscous effects, and boundary layers form. This can be explained by Stokes’s second problem, which suggests that the fluid shear next to SNs increases with the square root of frequency, $\sqrt{f}$. For CNs, the channel flow profile also depends on the frequency, adding a third layer of filtering. Astonishingly, the combinations of frequency filtering result in a constant sensitivity to flow velocity in SNs of zebrafish (*Danio rerio*) [89] and a constant sensitivity to flow acceleration in CNs of ruffe (*Gymnocephalus cerua*) [90] for a wide range of frequencies. This indicates that SNs and CNs are indeed suitable for sensing velocity and pressure, respectively.

Flow sensing of the lateral line systems enables behaviours such as prey detection, obstacle avoidance and rheotaxis [81, ch. 3]. Early studies focused primarily on how lateral line systems sense perturbations from small prey. Nocturnal predators such as the Lake Michigan mottled sculpin (*Cottus bairdi*) rely heavily if not exclusively on the lateral line [91,92]. Species that typically use vision to locate prey, such as bluegill sunfish (*Lepomis macrochirus*) [93], largemouth bass [94] and blacktip sharks [95] can still detect prey without vision as long as the prey is within half a body length away. Surface-feeding fish use lateral line systems to detect the pressure variation caused by prey at the water surface. Blind cavefish *Astyanax mexicanus* rely on hydrodynamic sensing to follow walls [96,97], avoid obstacles [72] and detect prey [88]. Sensing flow allows fish to hold their position either in free streams as they orient towards the incoming flow [98,99] or in regions behind an obstacle [100–102].

More recent studies have focused on the role of hydrodynamic sensing in dynamic manoeuvres. Kármán gaiting describes a synchronized movement between a fish and the vortex street behind an obstacle drag wake [103,104]. In such a gait, fish slalom in between the shed vortices to take advantage of the propulsion created by the fluid pressure gradient. This behaviour is only possible when fish can access the local hydrodynamic information [100]. Sensing vortex streets also allows European catfish (*Silurus glanis*) to follow the hydrodynamic trail of their prey [105,106]. Recently, vortex-matching has been identified as a coordination strategy for fish in pairs [46,107,108]. Surprisingly, it was concluded that neither vision nor lateral line systems are required for such behaviour [108], suggesting that it can arise from hydrodynamic interactions alone.

Only a few studies exist on the role of hydrodynamic sensing during schooling. Classical experiments were conducted in the late 1970s. Pitcher & Patridge investigated how a fish in a school behaves differently when it is deprived of vision or fluid sensing capabilities [25,109]. They showed that a blindfolded fish can still school, but with a different structure [109]. When vision is deprived, the fish prefer to stay closer to their neighbours. They concluded that social attraction, i.e. the active strategy to cohere as a group, is mediated by vision, whereas collision avoidance is mediated by lateral line information. Recent studies on firehead tetras (*Hemigrammus bleheri*), yellow-eyed mullets (*Aldrichetta forsteri*) and giant danios (*Devario aequipinnatus*) also showed that lateral line ablation disrupts schooling [110–112]. Without lateral line information, fish align less and collide more with other fish [110,111]. More recently, researchers demonstrated that while vision is sufficient for schooling in rummy-nose tetras (*Hemigrammus rhodostomus*) as they burst and coast in still water [113,114], the lateral line systems in giant danio (*Devario aequipinnatus*) is crucial for tail synchronization [115]. Experimental evidence also demonstrated that giant danio (*Devario aequipinnatus*) can form coherent schools in a dark room [116] and coordinate with a flapping hydrofoil or a robot that produces fish-like wakes [117,118]. It appears unquestionable that a certain level of schooling can be achieved in the absence of visual cues.

In addition to vision, inner ear hair cells sense the acceleration of the animal and the proprioceptors in muscles and connective tissues detect body deformation and fin deflection. These sensory modes provide supplemental information on the fluid environment that fish may use for coordination. Future animal experiments are required to elucidate the role of lateral line systems in schooling behaviour.

## 4. Flow-sensing in underwater robots

Inspired by lateral line systems, engineers have developed various electronic platforms that sense flows. These mechanical systems provide powerful tools to study fluid-mediated coordination in both natural and artificial systems. Fluid flow around fish schools depends only on the boundary conditions. Therefore, by mimicking the shape and movement of fish, a robot can reproduce the same wake, and sensors placed at locations of real fish’s lateral line systems will receive the same hydrodynamic information a fish would access. Hypotheses about how fish coordinate with each other can then be tested. Furthermore, incorporating flow sensing into underwater robot swarms allows artificial systems to adopt fish behaviour such as rheotaxis, obstacle avoidance and coordination.

On the smallest scale, lateral line systems inspire the designs of miniature flow sensors called ALL (artificial lateral line) sensors [119–122]. Taking advantage of recent advances in MEMS and NEMS (micro- and nano-electro-mechanical systems) manufacturing, new designs of flow sensors mimic structures of SNs and CNs and often include a single or an array of micro-pillars. Micro-pillars bend under fluid flow much like SNs, and piezoresistive, piezoelectric, capacitive or optical components can be used to convert mechanical deformation into electrical signals [120]. Since the same mechanism was used, these SN-mimicking sensors also respond differently to stimuli at different frequencies. In the past few years, there has been an increasing interest in developing CN-inspired sensors that measure the pressure difference between canal outlets [40]. Like in fish, artificial CNs are more suitable compared to artificial SNs when the boundary layer is thin. Protected by microfluidic channels, CN-inspired sensors are also less prone to damage. While ALL sensors closely mimic the mechanisms of lateral line systems and are customizable, they are difficult to manufacture and often require additional tuning and calibration. Therefore, most existing designs of autonomous robots use commercial flow sensors instead of ALL.

**Figure 5.**
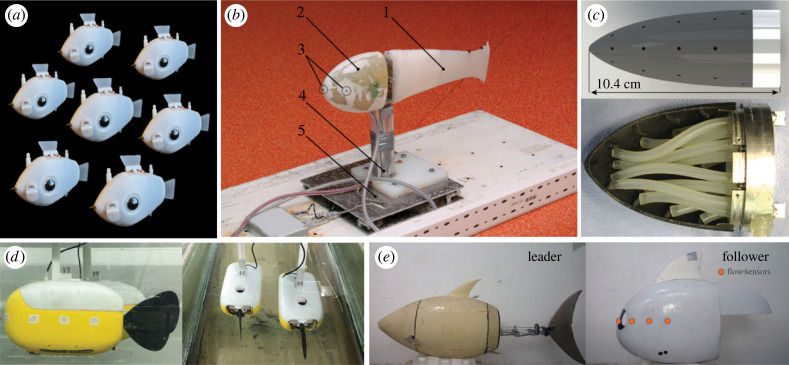
Underwater robots with onboard sensing capabilities. (*a*) Vision-based coordination in BlueSwarms, reproduced with permission from [37]. (*b*–*e*) Individual robots with flow sensors (permission from [123] for *b*, and [124] for *c*). (*d*,*e*) Robots that use flow-sensing to detect their neighbours (permission from [125] for *d*, and [126] for *e*).

Beyond bio-mimicry, robotics has emerged as a powerful tool for generating and testing biologically relevant hypotheses. BlueSwarm is the first autonomous underwater robot swarm capable of decentralized coordination (figure 5*a*) [37]. It demonstrates that robot fish can display milling behaviour by using vision alone. Nevertheless, the robots are limited to still water and slow speeds, where flow sensing may play a less significant role. One of the earliest robot fish with hydrodynamic sensing capabilities is the prototype under project FILOSE (Robotic FIsh LOcomotion and SEnsing) [123,127–130]. In 2011, Kruusmaa *et al*. reported how a robot fish could use one single pressure sensor to control its tail-beat frequency to keep up with the flow speed in the water channel [123] (figure 5*b*). By incorporating a controller inspired by the Braitenberg vehicle, this robot can perform rheotaxis by sensing the pressure difference between the two sides of its head [128]. With all five pressure sensors activated, the robot fish can differentiate between the flow behind an obstacle, i.e. Kármán vortex street, and an unobstructed laminar flow. In both cases, it is capable of exhibiting station-holding behaviour using hydrodynamic sensory information [130].

Sensing robots without fish-like beating tails benefited from a higher signal-to-noise ratio and enabled theoretical analyses. Researchers have increased the number of pressure sensors from 9 [124,131], 11 [132,133] to 20 [134] (figure 5*c*), making them effective flow sensing probes for measurements in the field [132,133]. The robotic platforms developed in [135–137] also lack actuated tails. Using a pair of Joukowski foils constrained to planar translation and pitching, researchers implement model-based control for the follower to perform Kármán gaiting [137]. Untethered robots can also benefit from sensing the fluid environment. Flow sensing enables free-swimming robots to estimate the robot’s own swimming speed and direction [138,139] and to follow the wall like a cavefish [140,141]. An eel-like underwater robot has been developed to coordinate its body undulations based on hydrodynamic feedback [142].

Flow sensing has also been used in robots to gather information about their neighbours. For a pair of robots towed in a flume, the follower robot can use the sensed hydrodynamic pressure variations to estimate the states of the leader robot, including its relative location, orientation, oscillating frequency, amplitude, etc. (figure 5*d*) [125,143]. A free-swimming robot can also locate its leader (figure 5*e*) [126]. The estimation of the neighbour’s location is more accurate when the neighbour is in front of the follower compared to when the neighbour is beside or behind the robot [126]. These studies have suggested that hydrodynamic information can indeed be used for coordination. Furthermore, they show the potential of using robots as a powerful tool for extracting hydrodynamic information and using it to achieve fish-like behaviour. However, free-swimming robots are rare and most studies are limited to only two sensing robots. Adding artificial lateral lines to platforms like BlueSwarms [37] remains an exciting future direction.

## 5. Challenges and opportunities

**Figure 6.**
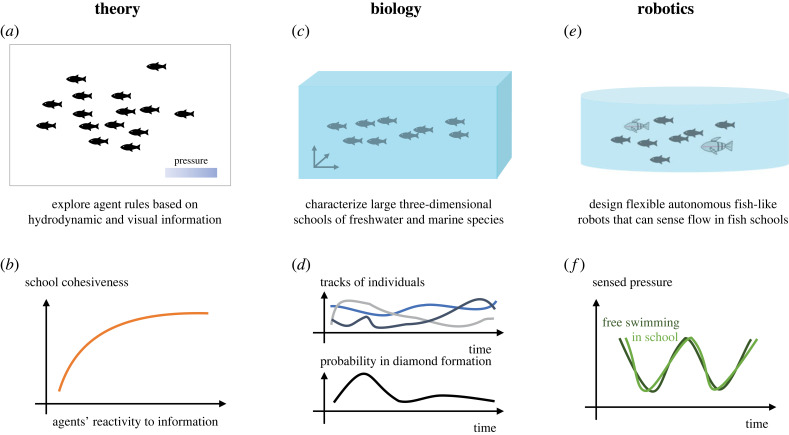
Future directions and goals for understanding underwater collectives using (*a*,*b*) theoretical, (*c*,*d*) biological and (*e*,*f*) robotic approaches.

Understanding underwater collectives requires knowledge from various disciplines. Research using live animals and robots presents two distinct philosophies for tackling complexity. In animal research, it is often difficult to control and isolate variables of interest, leading to an often messy but realistic picture. On the other hand, a biomimetic approach builds the complex system from the ground up and is excellent for studying each contributing factor in isolation. However, constructing these underwater systems is challenging and the fidelity of bio-mimicry is always compromised to some extent. Future studies that join different approaches would reveal novel insights into underwater collectives (figure 6).

### 5.1. Building realistic models of swarms

Agent-based models are critical for exploring and understanding collective phenomena, but the dynamics of the fluid environment has often been ignored. While this may be valid for vision-dominated animals such as starlings [144–147], underwater organisms have to interact with much larger fluid forces and the visibility of their environment is often lower. To investigate underwater systems, it is essential to couple agent-based models with computational fluid dynamic simulations (figure 6*a*). Such a coupled model will capture the realistic interaction between agents and their fluid environments: agents’ movements disturb the fluid field, and the fluid in turn propels the agents. Incorporating fluid field simulations also creates the opportunity to model agents’ behavioural rules based on their fluid surroundings. Such sensory-based multi-agent models will enable studies of how agents react to hydrodynamic cues, and how variations of this feedback mechanism affect the cohesiveness of the school (figure 6*b*).

The most glaring challenge with coupling collective behaviour to a flow solver is the computational cost. Current advances in computational fluid dynamics allow resolutions of fine details in flow structures. However, high-fidelity algorithms require a considerable amount of computational resources and time. One direct numerical simulation of a fish school swimming for a few seconds can take weeks to complete on a computer cluster. To reduce the computational cost, most existing fluid simulations of fish schools prescribe the fish’s movements and ignore the force balance around each individual and Newton’s second law. If the prescribed conditions such as the formation, tail-beat amplitude and frequency are inherently unstable, the formation cannot be sustained and any solutions reached would not be representative of biological collectives.

To ensure a force balance around individual agents while keeping computational cost low, the fidelity of the simulations must be compromised. This can be done by using potential flow theories [50,148]. However, since agents under these assumptions do not leave periodic wakes, the treatment is only applicable for sparse underwater collectives. An alternative method is by directly modelling the vortex street behind a two-dimensional swimmer [149]. In addition, adaptive mesh refinement and simplified agents have been used to conduct a parameter study of insect swarms under airflow [33]. Another promising approach that does not require significant sacrifice in flow field accuracy is to prescribe the trajectories and kinematics tracked from animal experiments. This approach will ensure that the simulation satisfies physical constraints automatically. The force balance around individuals in the simulation will be left as a check for numerical accuracy. Striking a balance between the computational cost associated with fluid simulation and reality (deformable body, unconstrained movement, large number of individuals, behavioural complexity) is critical for future research.

### 5.2. Characterizing collective movements through experiments

One fundamental challenge of studying fish schools is the difficulty of conducting animal experiments. Keeping, experimenting with, and tracking fish schools in three dimensions even in a laboratory environment requires expertise in both biology and computer vision (figure 6*c*). Most existing experiments used small freshwater fish (a few centimetres in length) in shallow aquaria, forcing formations to be planer which made tracking manageable [55,150]. While much progress has been made in animal pose tracking algorithms using approaches such as contrast-based segmentation [151–153] and machine learning [154–156], their performance in tracking dense fish schools remains less than ideal. Using one of the most popular tools in multi-animal tracking in DeepLabCut, tracking near planar shoals of silversides *Menidia beryllina* yields at best 70% mean average precision (mAP) [155]. For schools in three dimensions, fish frequently overlap with each other in any given video view. Isolating individuals from overlapping clusters requires ingenious solutions. Future tracking algorithms may benefit from using multiple cameras and stereo vision to simultaneously acquire a depth map from the camera [52,115], or from incorporating knowledge of fish swimming kinematics which have been reported to be generally similar across a diversity of fish families [157]. The recent development of large models for general computer vision such as [158] also provides tremendous opportunities for three-dimensional tracking fish schools.

To gain a deeper understanding of underwater collective behaviour, studies of a wider range of species are also critical [159]. Our knowledge of marine species that migrate in enormous groups, such as tuna and sardines, remains limited. Field experiments and novel approaches are essential. Using a towed net, a recent comparative study examined the swarming patterns of California market squid (*Doryteuthis opalescens*) and Pacific sardine (*Sardinops sagax*), which have distinct propulsive mechanisms [160]. Using rectified videos, another team of researchers measured the duration and size of fish shoal disturbances [161]. Adaptive resolution imaging sonar (ARIS) [162,163] and drone footage [164–167] have also emerged as powerful tools in identifying schooling structures in the field.

Studies of underwater swarms can also benefit from rigorous characterizations of their spatial–temporal features. Many influential studies that shaped our understanding of schooling formations use experiments that are based on trials under a minute long [55,59]. At this timescale, the habituation of the animals and the initialization of flow field may both affect the schools’ structures. Recordings that are around an hour long allowed researchers to apply statistical methods and analyse the state of the school [150,168]. Even longer recordings that are multiple hours or even days long are necessary to fully characterize fish school formations and answer fundamental research questions: How frequent are fish schools in certain formations (figure 6*d*)? How stable are these formations? It will be interesting to see if other patterns of coherent movement will emerge from systematic studies like these.

Tracking fish schools provides a crucial step toward understanding their collective behaviour. Since the flow field depends only on the movement of the school, it can be reconstructed using fluid simulations or robotic experiments once the tracks are obtained. Through such hybrid approaches, one can obtain detailed flow information such as the fluid stress and pressure field next to each individual’s lateral line systems without directly experimenting with the animals. In addition, other sensory information such as vision and proprioception may also be inferred. Without further measurements, it will be tangible to derive a mapping from what a fish may sense to its behaviour. Note that both the input and output spaces of this mapping are large and the relationship may be nonlinear. Searching for low-dimensional structures from high-dimensional data is a recurring theme in biophysics [169]. Dimension-reduction techniques such as principal component analysis and proper orthogonal decomposition may be used [170,171]. Statistical methods such as correlation functions and transfer entropy may also be applied [117,172]. Furthermore, future research may adopt cutting-edge model-discovery approaches such as sparse identification of nonlinear dynamical systems (SINDy) [173] and knowledge-based neural ODE (KNODE) [174,175] to infer the behavioural strategy of schooling fish. Application of these methods on thousands of tracks in a large school is an exciting future direction.

### 5.3. Designing flow-sensing robot collectives

Bioinspired robots enable rigorous studies of hydrodynamic effects on underwater swimmers, isolating factors related to animal behaviour. While tethered robots and hydrofoils provide better control for experimental work, they prevent natural movements that arise from hydrodynamic interactions. Air bearing systems allow a tethered object to move freely in two dimensions, but movements in the other four degrees of freedom are constrained. Critically, the pitch angles of hydrofoils are often controlled. In fact, rigid hydrofoils can only generate thrust by pitching, which is driven by the motor it is attached to. This is distinct from fish, who generate thrust by contracting their muscles and *deforming* their body. In the absence of external control, free-swimming robots have to generate thrust through deformation and thus move more like real fish [37,176–178]. It has also been discovered that tuning flexibility is essential to match the kinematics of fish [177,179–183]. Future studies that deploy groups of autonomous free-swimming robots may lead to discoveries of metastable schooling configurations that cannot be observed with tethered hydrofoils.

Bioinspired robots also provide unique opportunities to peek into the sensory world of biological organisms. If the morphology and kinematics of fish are mimicked, a fish robot in a swarm will sense the same fluid pressure and shear that a fish in such a school must sense. While electronic sensors may differ from neuromasts in their sensitivity and range, the hydrodynamic information accessible to them is identical. Adding arrays of flow sensors to systems like BlueSwarm [37] would reveal detailed information about the flow field around the fish robot. Hybrid experiments that swim fish robots among living fish school will provide insights into the roles of sensing in coordination and school formation control (figure 6*e*,*f*). With the added flow-sensing capabilities and knowledge of fish’s hydrodynamic feedback algorithm, future robots may be able to perform vortex matching and increase energy efficiency. Development of miniature artificial lateral line sensors that are scalable and easily deployable will also significantly advance our understanding of the hydrodynamic information embedded in the environment.

## 6. Concluding remarks

Fish schools are mesmerizing. They have inspired people from all walks of life and are an excellent example of collective behaviour. They are also easy to observe; most pet stores sell dozens of tropical species that readily school in aquaria. Despite the ubiquity, it remains mysterious how they coordinate with each other so well and how fluid environment dictates their collective behaviour.

To some, the mystery may seem to have been solved when the Vicsek and Boids models showed that by keeping a certain distance from their neighbour, a flock of agents could avoid obstacles and move cohesively. Nonetheless, these models overlooked limitations of sensing in biological organisms. The range, resolution and speed of information acquisition are all limited; an animal cannot perfectly estimate its neighbours’ locations and their distances from it. This gap in our understanding of swarms becomes glaring as roboticists struggle to translate the social force models to robots—robots also have limited sensory capabilities.

In bridging the gap, interdisciplinary collaboration is essential. Fluid mechanics researchers have long pondered the fluid–structure interactions of hydrofoils; biologists have meticulously described the morphology and sensing mechanism of lateral line systems; roboticists can now assemble fleets of underwater vehicles. To gain deeper insights into underwater coordination, we must gather a diverse team of experts from different backgrounds.

Finally, we look forward to applications of *fluid stigmergy* in other collective systems where signals are transported by dynamic fluid fields. In fact, many organisms from microscopic to macroscopic coordinate with each other using chemical signals that are suspended in fluids like water or air. The role of fluid flow in these collectives remains to be investigated.

### 6.1. Citation diversity statement

This statement is inspired by a recent initiative [184]. The accumulated knowledge in the topic reviewed here joins not only a diversity of disciplines but also contributions from researchers of different genders and ethnic origins. Recent work revealed that women and other minority scholars are under-cited even as factors representing seniority and productiveness are accounted for (see [185] for a detailed analysis). Here, we provide a breakdown of our references using the tool https://github.com/dalejn/cleanBib. Our references contain 8.2% woman (first author)/woman (last author), 12.0% man/woman, 18.6% woman/man and 61.2% man/man. By race, our references contain 14.8% author of colour (first)/author of colour (last), 6.0% white author/author of colour, 18.0% author of colour/white author, and 61.2% white author/white author. Following the original method, a colour author can be Asian, Hispanic, or Black. Of all 366 first/last authors (counting duplicates), 73.2% are white, 22.4% are Asian and only 4.4% are Hispanic and Black combined. This method is limited in that the predictions made by gender-api.com are not always correct. We have found that for a few authors of Asian origins, gender-api.com predicted the incorrect gender while claiming to have a near-perfect confidence score. We manually corrected a few predictions upon looking up researchers’ profiles, but the results cannot be assumed to be completely accurate. The statistics we report fail to include intersex, non-binary, transgender people, indigenous and mixed-race authors, or those who may face differential biases due to the ambiguous racialization or ethnicization of their names. Under-representations of other dimensions such as sexual orientation, (dis)ability, class and their intersections are difficult to reveal. We look forward to future work that could help us better understand how to support equitable practices in science.

## Data Availability

This article has no additional data.
